# A case report on acquired reactive perforating collagenosis

**DOI:** 10.1097/MD.0000000000039071

**Published:** 2024-07-19

**Authors:** Yuanting Su, Wei Cui

**Affiliations:** a Department of Dermatology, The Second People’s Hospital of Hefei, Hefei, Anhui, China.

**Keywords:** acquired reactive perforating collagenosis, case report, elastosis perforans serpiginosa, skin microscopy, tumors

## Abstract

**Introduction:**

Acquired reactive perforating collagenosis (ARPC) is a rare perforating skin disease with unclear pathogenesis, often leading to misdiagnosis. Utilizing noninvasive skin microscopy improves diagnostic accuracy while reducing misdiagnosis rates.

**Patient concerns:**

Given its association with systemic diseases, comprehensive examinations are crucial for early detection of related diseases such as tumors. Clinically, it still lacks standardized guidelines for the treatment. Clinical treatment is mostly based on symptomatic treatment. Oral administration of pregabalin capsules can significantly relieve itching symptoms, and narrow-wave ultraviolet irradiation can accelerate the recovery of skin lesions.

**Diagnosis:**

Dermoscopy and skin biopsy was used to confirm this case was ARPC.

**Interventions:**

Treatment was based on oral administration of 20 mg prednisone, 1 tablet of loratadine, 1 tablet of pregabalin in the morning and evening, and external application of halomethasone ointment.

**Outcomes:**

Itching symptoms were significantly relieved.

**Conclusion:**

This case report demonstrates that clinical dermoscopy can improve the diagnosis rate of ARPC, and pregabalin capsules can significantly relieve itching symptoms.

## 1. Introduction

**Figure 1.**
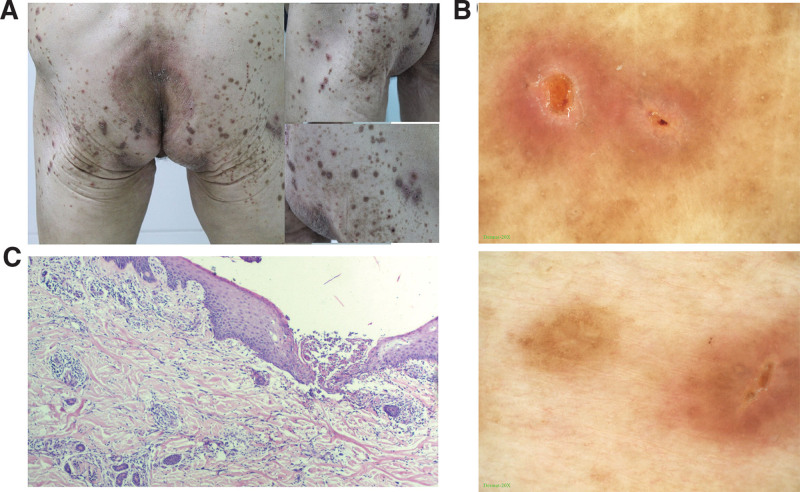
(A) Back view (left) and side view (right) of the patient’s partial lesions. (B) Dermoscopy examination. (C) Skin histopathological examination.

The patient, a 54-year-old female, presented with papular nodules on the waist, buttocks, and bilateral thighs accompanied by intense itching for over 6 months. Her medical history included 30 years of hypertension, 20 years of coronary heart disease, 4 years of diabetes, uterine fibroid removal, and allergies to penicillin and phosphorus drugs. Cutaneous examination revealed scattered papules and nodules on the waist, buttocks, and bilateral thighs with central umbilication necrosis or keratin plug, along with scratch marks and ulcers, accompanied by Koebner phenomenon (Fig. 1A).

Laboratory tests: no obvious abnormalities were found in routine blood tests, liver and kidney function, blood sugar, blood lipids, tumor indicators, electrocardiogram, and chest CT.

Dermoscopy examination: central yellow-brown structureless area, white edge around the blood spot or collar-like scales around the blood spot, peripheral pink structureless area and punctate blood vessels (Fig. 1B, top); central yellow-brown bleeding Scab, surrounded by concentric brown and white edges; central white structureless, surrounded by curves and brown mesh lines (Fig. 1B, bottom).

Skin histopathological examination: cup-shaped depression in the epidermis, columnar keratotic plugs can be seen, including parakeratotic cells, keratinized epithelium and denatured collagen fibers; the epidermis at the bottom of the cup-shaped depression has atrophied and thinned, and basophilic denatured collagen fibers can be seen from the dermis upward. After penetrating the epidermis, scattered inflammatory cell infiltration can be seen around the superficial blood vessels in the dermis (Fig. 1C).

Dermatoscopy and skin biopsy confirmed this case was acquired reactive perforating collagenosis (ARPC). Treatment was based on oral administration of 20 mg prednisone, 10 mg loratadine, 75 mg pregabalin in the morning and evening, and external application of halomethasone ointment. After 1 week of treatment, the itching was significantly relieved, the skin lesions were dim in color, and there was no new rash; After 1 month of treatment, the rash basically subsided, with a little pigmentation.

## 2. Discussion

The etiology and pathogenesis of ARPC are still unclear. It is common in adults and is often associated with kidney disease, diabetes, liver disease, autoimmune disease, thyroid disease, zonal disease, herpes, scabies infection, malignant tumors and other diseases.^[1]^ Studies have shown that there is a potential correlation between microvascular disease in diabetes and ARPC.^[2]^ Diagnostic criteria include cup-shaped depression in the epidermis, with basophilic collagen fibers discharged through the epidermis; concave-shaped papules or nodules in the umbilicus, with a corneal plug in the center; age > 18 years old. Because the diagnosis of this disease relies on pathological biopsy, it is easily misdiagnosed during the first clinical diagnosis. According to literature reports, the misdiagnosis rate reaches 47% to 77%.^[3]^ The Koebner phenomenon goes unreported in most cases. The extensor sides of the limbs are more common, but the trunk and face may also be affected. The back is an uncommon site, and the lesions in this patient were mainly concentrated on the lower back and buttocks. In the absence of histopathological biopsy, dermoscopy can be an effective method for rapid diagnosis of ARPC.^[4]^

There are 3 common features under dermoscopy. First, the center of the lesion is a tan structureless area, which histopathologically corresponds to keratin fragments in the center; the white structureless area in the center may correspond to the dermis in histopathology of collagen fibers. Secondly, the white edges and collar-like scales of different thicknesses on the periphery may correspond to the invaginated hyperplastic epidermis. Third, there is a pink inflammatory circle on the outside, which is composed of short circular blood vessels on the central side and punctate blood vessels on the peripheral side, which may represent an inflammatory reaction in the superficial dermis with vasodilation; the surrounding brown mesh lines may correspond to high-pigmented of basal layer keratinocytes, the coexistence of these 3 characteristics facilitates early diagnosis. The dermoscopic manifestations of ARPC in this case were consistent with the above characteristics, and the subsequent pathology confirmed the diagnosis, indicating that noninvasive dermoscopy is beneficial to improving diagnosis and is worthy of use. Kim et al reported that scratching is one of the main reasons for the exacerbation of the disease, so controlling itching has become the primary purpose of treating ARPC.^[5]^ It is mentioned in the literature that glucocorticoids, allopurinol, isotretinoin, etc can be used for treatment. The mechanism may be to regulate epidermal keratosis, anti-inflammation, prevent collagen degeneration, etc. Additionally, physical therapies such as narrowband ultraviolet radiation b (UVB) and 308 nm excimer laser have been proposed.^[6]^ The patient in this case used pregabalin capsules to relieve itching, and the effect was obvious without obvious side effects, suggesting its clinical utility.

This case report presents a case in which ARPC can be diagnosed based on the patient’s symptoms, dermatological examination, and laboratory examination. With the development of pathological biopsy of skin tissue, the diagnostic rate of this disease can be greatly improved. In addition, in the absence of pathological biopsy, dermoscopy can be an effective method for rapid diagnosis of ARPC. After ARPC is diagnosed, other systemic diseases should be actively searched for, and potential extracutaneous diseases should be revealed through relevant auxiliary examinations. In most cases, the diagnosis of ARPC needs to be confirmed by characteristic histopathological signs, but multiple repeated biopsies may be required. The pathological signs are different at different stages of the disease. When biopsy cannot be performed or diagnosis cannot be made, dermoscopy can be widely used as an auxiliary noninvasive examination method.

During the diagnosis and treatment of this patient, the following issues are worthy of consideration: First, the patient had a history of hypertension, coronary heart disease, and diabetes, but his condition was stable after taking medication regularly, and no abnormalities were found in tumor indicators and chest CT and other related examinations after admission, suggesting that this patient There is no obvious correlation between the secondary skin disease and the original underlying disease; secondly, during the physical examination at admission, arc-shaped light red papules with clear borders were visible on the buttocks, with a dark center and covered with adhesive scales. At that time, it was mistaken for tinea cruris and no pathology was performed. After tissue biopsy and dermoscopy, when the results of fungal examination and fungal culture are negative, the diagnosis of fungal infection is ruled out, and the arc-shaped rash gradually subsides during treatment, we judge it to be creeping penetrating collagenosis. This case tells us When similar cases are encountered in clinical work in the future, fungal infection should be ruled out and dermoscopy and pathological tissue biopsy should be completed to avoid missed diagnosis and misdiagnosis. Finally, after the patient was admitted to the hospital, the itching was not significantly relieved after protest and antihistamine treatment. After the addition of pregabalin capsules, the itching was significantly relieved, and it can be widely used in clinical practice.

## Author contributions

**Data curation:** Yuanting Su, Wei Cui

**Funding acquisition:** Yuanting Su.

**Resources:** Yuanting Su.

**Supervision:** Yuanting Su.

**Validation:** Yuanting Su, Wei Cui.

**Writing – original draft:** Yuanting Su, Wei Cui.

**Writing – review & editing:** Yuanting Su, Wei Cui.
